# Point-of-care ultrasonography for the diagnosis and manual detorsion of testicular torsion

**DOI:** 10.1007/s10396-023-01374-z

**Published:** 2023-10-20

**Authors:** Takahiro Hosokawa, Yutaka Tanami, Yumiko Sato, Eiji Oguma

**Affiliations:** Department of Radiology, Saitama Children’s Medical Center, 1-2 Shintoshin Chuo-ku Saitama, Saitama, 330-8777 Japan

**Keywords:** Testicular torsion, Acute scrotum, Ultrasound, Sonography, Whirlpool sign

## Abstract

Testicular torsion is a urological emergency caused by the loss of testicular tissue due to ischemic damage. Rapid diagnosis and urgent treatment play a crucial role in the management of testicular torsion. Manual detorsion can be performed at the bedside, thereby reducing the duration of ischemia. Recent studies have reported the use of point-of-care ultrasonography for diagnosing testicular torsion; however, no review article has focused on the ultrasonographic findings pertaining to manual detorsion. This review describes the diagnosis of testicular torsion and the ultrasonographic indications for manual detorsion. Spermatic cord twisting or the whirlpool sign, absence of or decreased blood flow within the affected testis, abnormal testicular axis, abnormal echogenicity, and enlargement of the affected testis and epididymis due to ischemia are the sonographic findings associated with testicular torsion. The following findings are considered indications for manual detorsion: direction of testicular torsion, i.e., inner or outer direction (ultrasonographic accuracy of 70%), and the degree of spermatic cord twist. The following sonographic findings are used to determine whether the treatment was successful: presence of the whirlpool sign and the degree and extent of perfusion of the affected testis. Misdiagnosis of the direction of manual detorsion, a high degree of spermatic cord twisting and insufficient detorsion, testicular compartment syndrome, and testicular necrosis were found to result in treatment failure. The success of manual detorsion is determined based on the symptoms and sonographic findings. Subsequent surgical exploration is recommended in all cases, regardless of the success of manual detorsion.

**Supplementary Information:**

The online version contains supplementary material available at 10.1007/s10396-023-01374-z.

## Introduction

Acute scrotum can result from various etiologies, such as testicular torsion, inflammatory disease, and testicular trauma [1, 2], and the treatment strategy varies based on these etiologies. Testicular torsion is a urological emergency, and urgent intervention is necessary to prevent testicular necrosis [3]. Inflammatory diseases require adequate medical treatment [2]. Thus, rapid and accurate diagnosis is important for acute scrotum, and ultrasonography is widely used for diagnosing acute scrotum in pediatric patients [1, 4, 5].

Occurrence of testicular necrosis depends on the duration of symptoms and the degree of spermatic cord twisting in pediatric patients with testicular torsion [6]. Therefore, rapid detorsion is critical, and rapid diagnosis and treatment via manual or surgical detorsion play a crucial role in recovering testicular blood flow [7–9]. Unlike surgical detorsion, manual detorsion can be performed immediately after diagnosing testicular torsion, thereby decreasing the duration of testicular ischemia [7, 10–16]. Reports on the use of point-of-care ultrasonography for the diagnosis of testicular torsion have increased in recent years [7, 12, 13, 16–22]; however, no review article has described its use for manual detorsion and determined its success rates.

This review aimed to summarize the ultrasonographic findings used for the diagnosis of testicular torsion and the ultrasonographic indications for manual detorsion.

### Ultrasonographic findings for the diagnosis of testicular torsion

Testicular torsion is usually diagnosed based on the presence of the following ultrasonographic findings [23–27]:

1. Presence of spermatic cord twisting or the whirlpool sign

**Fig. 1:**
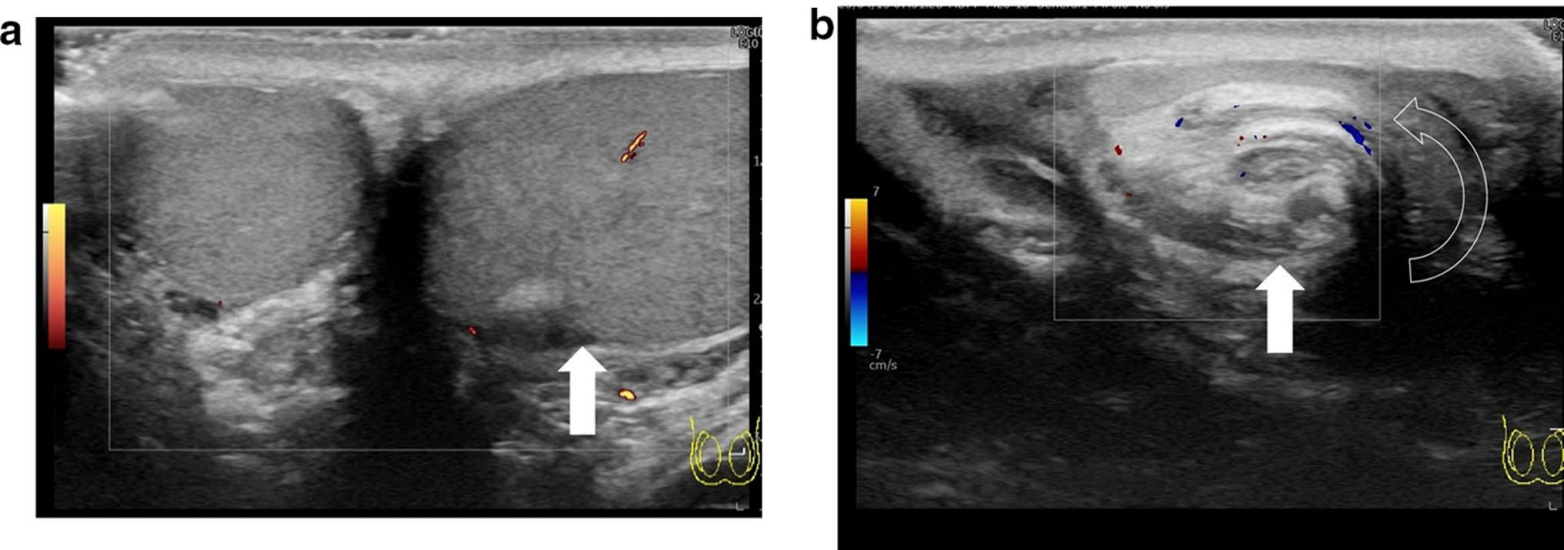
Ultrasonographic images of a 14-year-old boy with left testicular torsion. **a** The left testis is swollen, and the axis of the left testis is abnormal compared with that of the right testis. **b** The whirlpool sign is evident. Movie before manual detorsion; ultrasonogram shows the inward twisting of the left spermatic cord (curved arrow). The spermatic cord shows 360° twisting. Manual detorsion is performed via outer direction based on this finding. Movie after manual detorsion; ultrasonogram shows that the twisting of the spermatic cord is resolved, and redundant spermatic cord (arrow) is evident. A pseudomass (arrowhead) composed of congested epididymis, proximal vas deferens, and redundant vascular bundle is visualized after successful manual detorsion

**Fig. 2:**
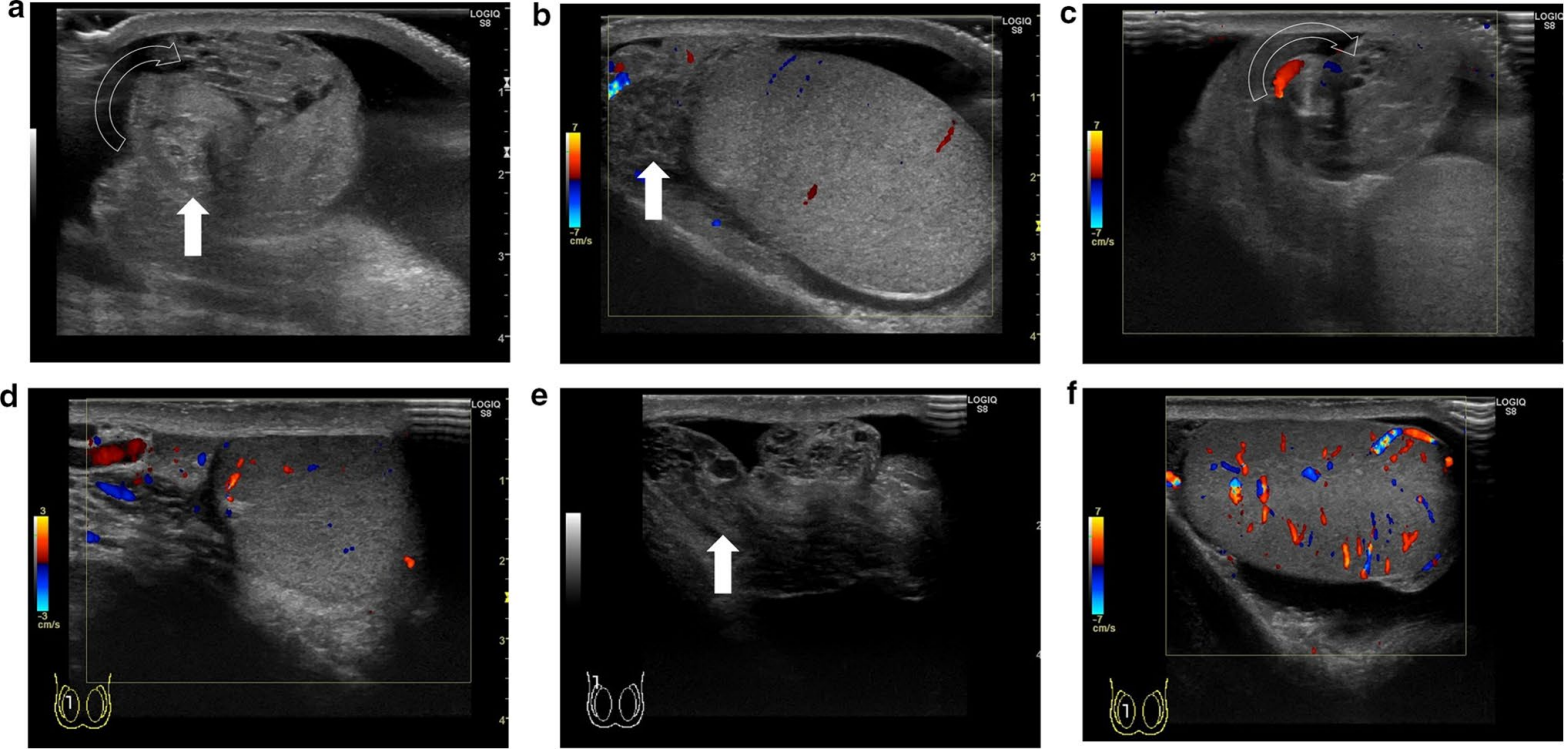
Ultrasonographic images of a 14-year-old boy with right testicular torsion. **a** The whirlpool sign is evident. **b** Swelling of the right testis and decreased vascular flow are evident. **c** The whirlpool sing is preserved after manual detorsion. **d** The vascularity within the affected testis is greater than that before manual detorsion, but it is not hypervascular. **e** The whirlpool sign is diminished after subsequent manual detorsion. **f** Hypervascularity within the testis is evident

The whirlpool sign is defined as the twisting of the spermatic cord at the external inguinal ring or scrotal sac [24, 25]. The presence of the whirlpool sign is a critical finding that is used to diagnose testicular torsion. Figures 1 and 2 show representative images of spermatic cord twisting or the whirlpool sign.

(2) A decrease or absence of blood flow within the affected testis

**Fig. 3:**
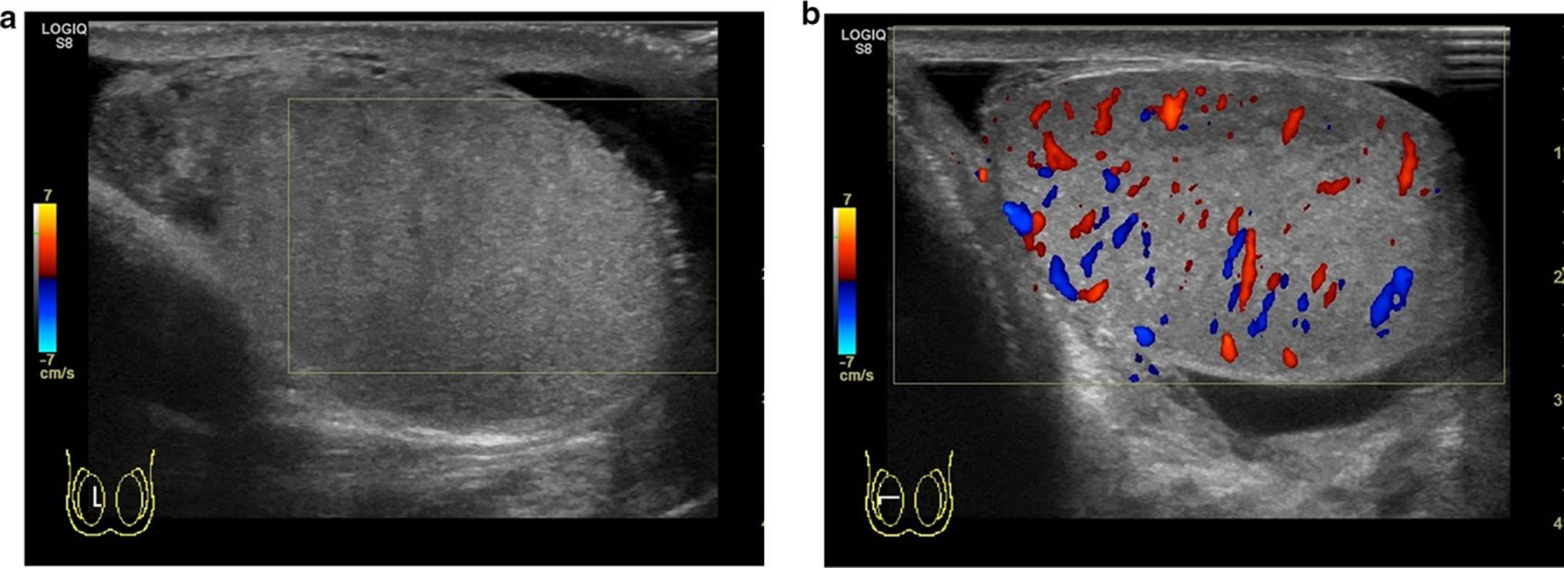
Ultrasonographic images of a 14-year-old boy with right testicular torsion. **a** Right testicular swelling is evident, and vascular flow is absent before manual detorsion. **b** Hypervascular flow is evident after manual detorsion

Absence of blood flow may be indicative of testicular torsion. However, blood flow within the affected testis may be preserved, with lesser perfusion observed within the affected testis than that within the unaffected side [7, 13, 24]. Thus, testicular torsion can occur even in cases with preserved blood flow within the affected testis. Figures 1, 2, and 3 show representative images of absence of or a decrease in the blood flow within the affected testis.

(3) Abnormal testicular axis

**Fig. 4:**
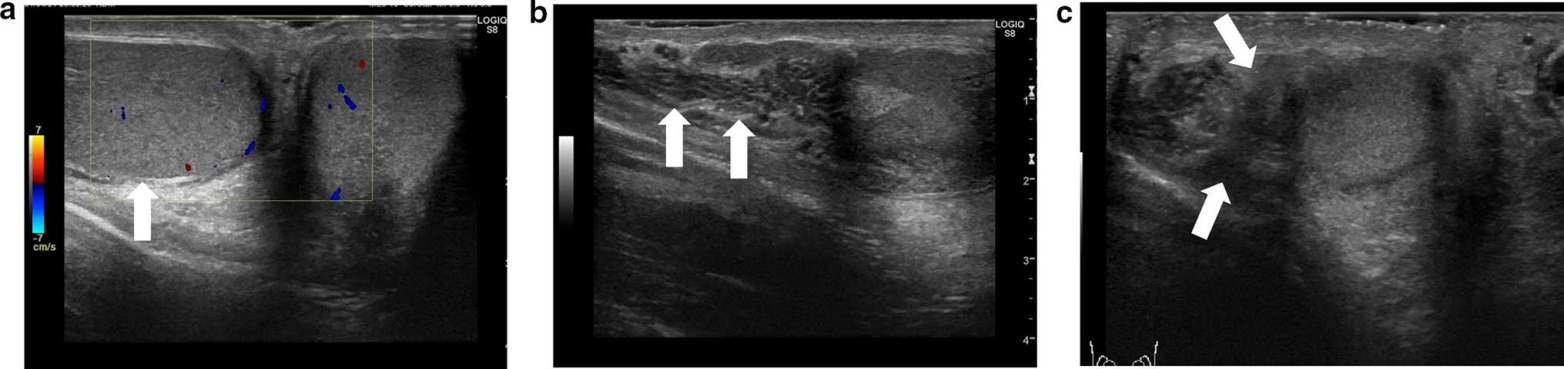
Ultrasonographic images of a 13-year-old boy with right intermitted testicular torsion. **a** The right testis has an abnormal axis and decreased blood flow. **b** The spermatic cord is redundant, but the whirlpool sing is absent. **c** A pseudomass is present adjacent to the testis. Intermitted testicular torsion was diagnosed based on these findings

The presence of bell-clapper deformity is a risk factor for testicular torsion [24]. Bell-clapper deformity is characterized by the lack of posterior attachment of the tunica vaginalis parietal lamina to the epididymis and an incomplete connection between the epididymis and testis, resulting in detachment of the epididymis from the lower testicular pole [24]. Intravaginal testicular torsion can occur due to the presence of bell-clapper deformity, and twisting of the spermatic cord can result in upward rotation of the lower testicular pole [13, 24]. The affected and unaffected testicular axes are asymmetrical and can be visualized as an abnormal horizontal or oblique line. Figures 1 and 4 show representative images of abnormal testicular axis.

(4) Abnormal echogenicity and enlargement of the affected testis and ischemia of the epididymis

Torsion can result in obstruction of venous blood return, which can lead to enlargement of the testis and edema [20, 24, 28–30]. Testicular necrosis may also occur in some cases, with the affected testis exhibiting heterogeneous echogenicity [16, 29–33]. Abnormal echogenicity can be detected by carefully comparing the echogenicity within the affected and unaffected testes. An enlarged epididymis without hyperperfusion must be differentiated from acute epididymitis [24, 34]. Figures 1 and 2 show representative images depicting abnormal echogenicity and enlargement of the affected testis and ischemia of the epididymis, respectively.

### Atypical cases and differential diagnosis

The following characteristics have been reported in atypical cases of testicular torsion [34–37].

1. Intermittent testicular torsion

Intermittent testicular torsion is characterized by the presence of acute unilateral scrotal pain that resolves spontaneously [34, 37]. The whirlpool sign may not be detected in cases of intermittent testicular torsion, and the testicular blood flow may be normal, which makes diagnosing this condition challenging [34]. The presence of a pseudomass, comprising congested epididymis, proximal vas deferens, and redundant vascular bundles, located below the spermatic twisting with hypervascularity is suggestive of intermittent testicular torsion [24, 34, 37]. Figure 4 shows representative images depicting intermittent testicular torsion.

(2) Perinatal testicular torsion

**Fig. 5:**
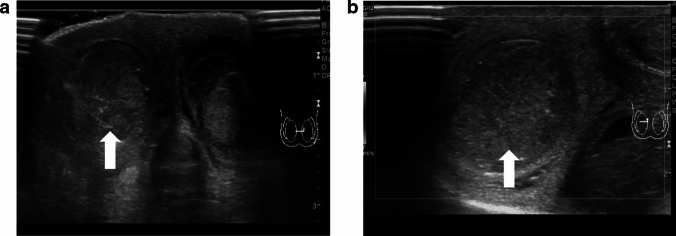
Ultrasonographic images of a 0-day-old neonate with right testicular torsion. **a** Swelling of the right testis is evident. **b** Heterogeneous echogenicity is observed within the affected testis. Testicular torsion and necrosis due to torsion were surgically diagnosed

Perineonatal testicular torsion is defined as testicular torsion that occurs during the gestational and neonatal periods [38–43]. Testicular salvage is usually difficult in these cases as the onset of spermatic twisting is unclear and surgical intervention is not possible [38, 39, 42]. The size and echogenicity of the testes are the factors influencing testicular salvage [30, 44–46]. Smaller size of the testes and heterogeneous echogenicity within the testes indicate the occurrence of testicular necrosis. Figure 5 shows representative images depicting perineonatal testicular torsion.

(3) Torsion of the undescended testis

**Fig. 6:**
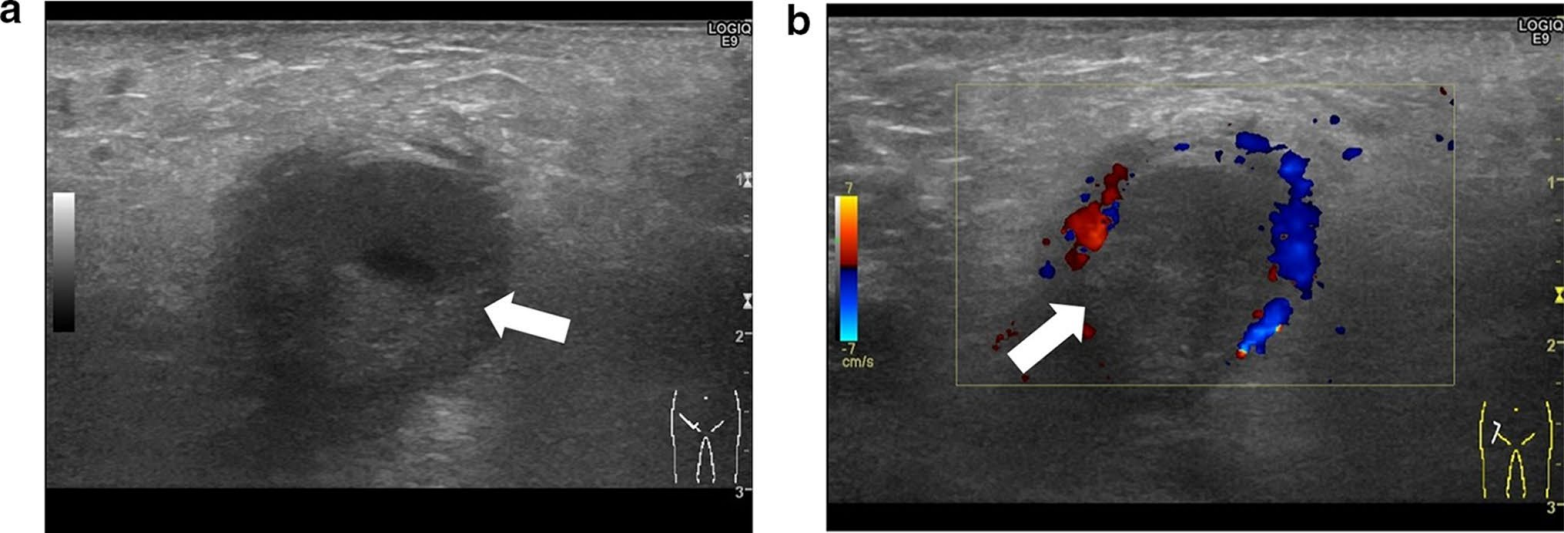
Ultrasonographic images of a 11-month-old baby with undescended testicular torsion. **a** Ultrasonography shows swelling of the inguinal soft tissue, and low-echoic testis is evident. **b** Color Doppler sonogram shows absence of blood flow within the undescended testis

Approximately 60% of patients with torsion of the undescended testis have comorbidities such as cerebral palsy or neuromuscular diseases [47]. This condition may be misdiagnosed as an incarcerated inguinal hernia [48]. Figure 6 shows representative images depicting torsion of the undescended testis.

Various diseases can result in the occurrence of acute scrotum, which can be differentiated from testicular torsion.

1. Acute epididymitis with/without orchitis

**Fig. 7:**
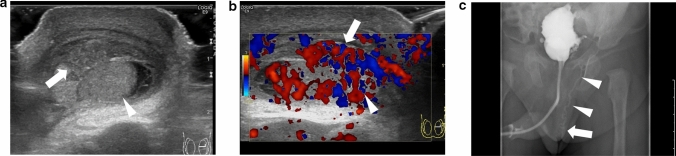
Ultrasonographic images of a 2-month-old baby with acute epididymitis with orchitis. **a** Swelling of the epididymis and edematous changes in the scrotal dermis are evident. **b** Hypervascularity of the epididymis and testes can be visualized. Epididymitis with orchitis was diagnosed based on these findings. **c** Voiding CUG shows reflux into the spermatic cord and epididymis

**Fig. 8:**
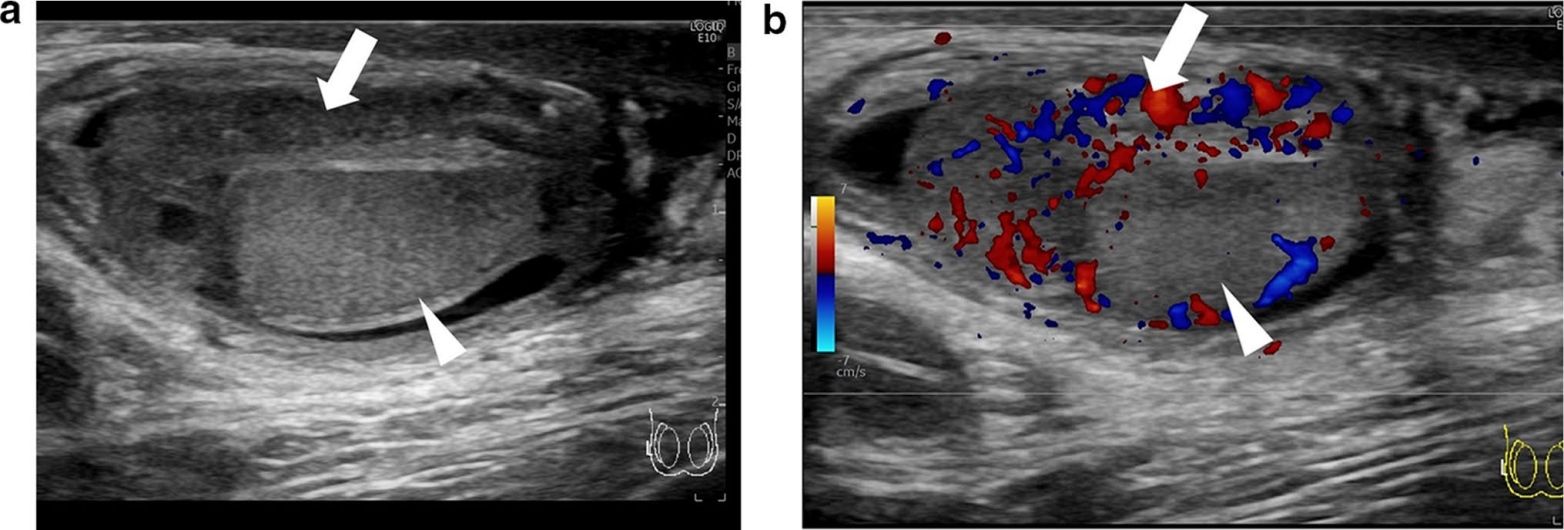
Ultrasonographic images of a 2-year-old with acute epididymitis without orchitis. **a** Swelling of the epididymis and edematous change in the dermis are evident. **b** Hypervascularity of the epididymis is evident. Acute epididymitis without orchitis was diagnosed based on these findings

Acute epididymitis is one of the most common causes of acute scrotum. Hyperperfusion of the epididymis can be used to diagnose acute epididymitis, which may be accompanied by orchitis in some cases. Anatomical abnormalities are often detected in the epididymis in such cases [4, 25, 49]. Arterial occlusion may also occur due to inflammation [50]. Figures 7 and 8 present representative images depicting acute epididymitis with and without orchitis, respectively.

(2) Segmental testicular infarction

**Fig. 9:**
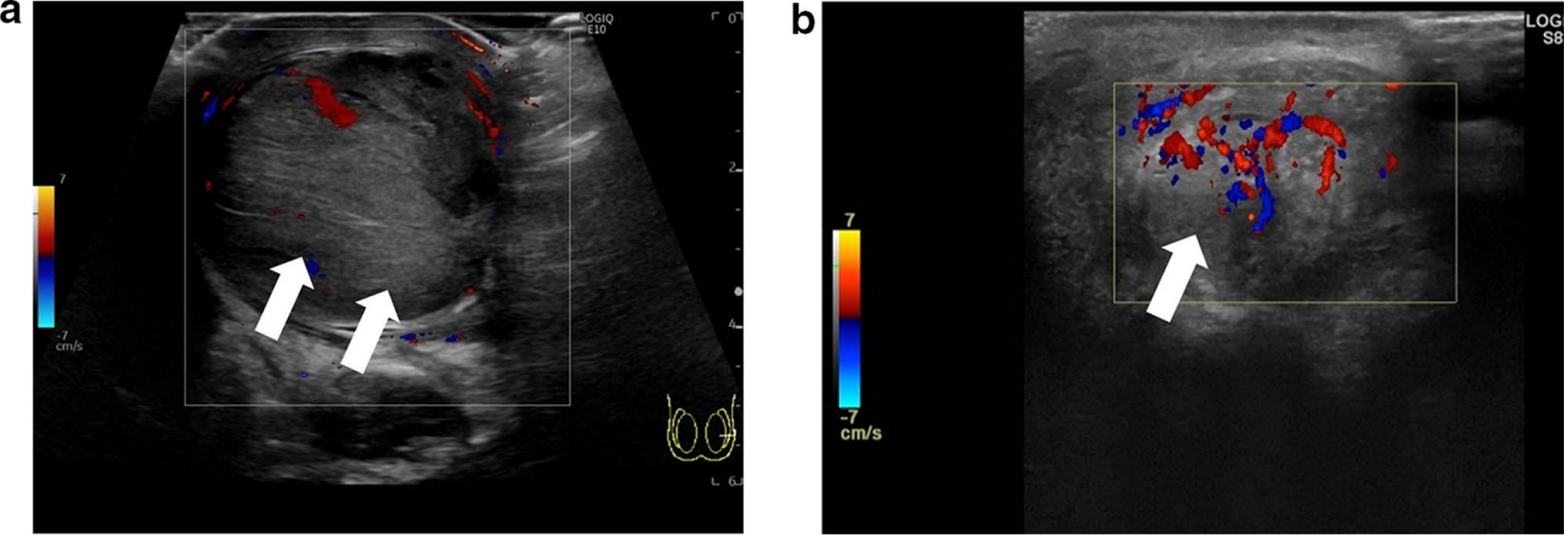
Ultrasonographic images of a 13-year-old boy with epididymitis accompanying segmental testicular infarction. **a** The upper pore of the right testis shows heterogeneous echogenicity. **b** The epididymis shows hypervascularity; therefore, epididymitis accompanying segmental testicular infarction was diagnosed

Segmental testicular infarction is characterized by acute scrotal pain in only one segment of the testis. The upper pole of the testis is the most frequently affected region, and normal testicular parenchyma is retained [31, 50, 51]. The ultrasonographic findings include a focal hypoechoic region at the upper pole of the testis without color Doppler flow in the hypoechoic region. Segmental testicular infarction usually occurs due to obstruction of arterial flow within the testes [31, 52]. The testes are supplied by the testicular artery, cremasteric artery, and artery of the ductus deferens [52]. Interruption of one of these arteries results in segmental infarction. Epididymitis, orchitis, trauma, sickle cell disease, or vasculitis may result in segmental infarction due to the interruption of these arteries [51]. Figure 9 shows representative images depicting segmental testicular infarction.

(3) Idiopathic scrotal edema or orchitis without systemic diseases

**Fig. 10:**
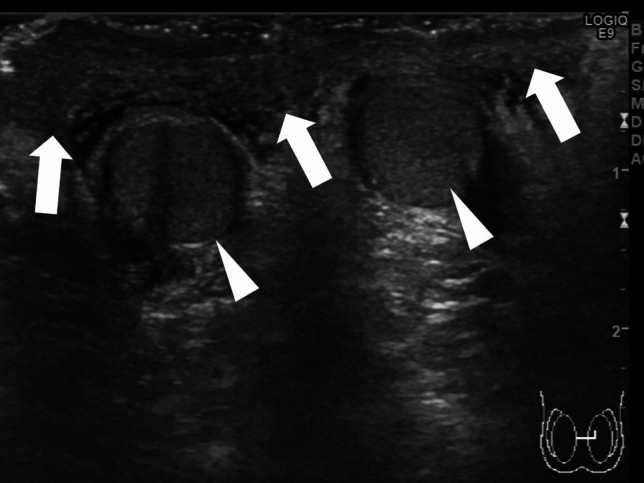
Ultrasonographic images of a 1-year-old boy with idiopathic scrotal edema. Low echoic changes due to edema in the dermis of the right and left scrota are evident. The patient was diagnosed with idiopathic scrotal edema based on these findings

Acute idiopathic scrotal edema is defined as a self-limiting thickening of the scrotal sac due to inflammation that usually resolves within 2–4 days [5, 53]. The characteristic ultrasonographic finding is marked by hypoechoic thickening of the scrotal sac with a normal epididymis [5, 49]. Approximately 50% of patients diagnosed with idiopathic scrotal edema are between the ages of 5 and 8 years; however, males under the age of 2 years and over the age of 12 years may also present with idiopathic scrotal edema [53]. Figure 10 shows representative images depicting idiopathic scrotal edema or orchitis without systemic diseases.

(4) Idiopathic scrotal edema, epididymitis, or orchitis associated with systemic diseases

**Fig. 11:**
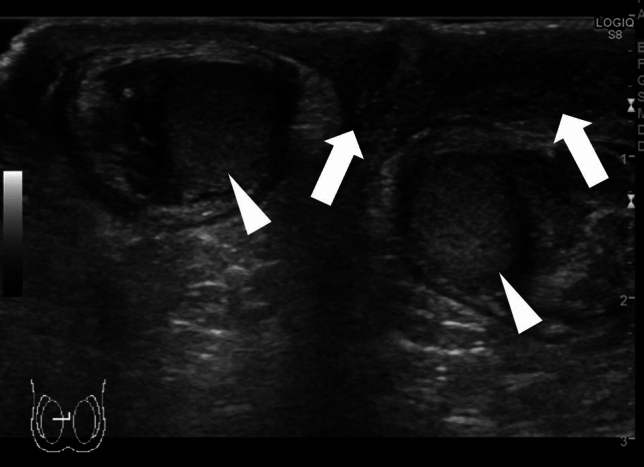
Ultrasonographic images of a 5-year-old boy with acute scrotum associated with IgA vasculitis. Edematous changes are evident in the dermis of the right and left scrota. Purpura was detected, leading to the diagnosis of IgA vasculitis

**Fig. 12:**
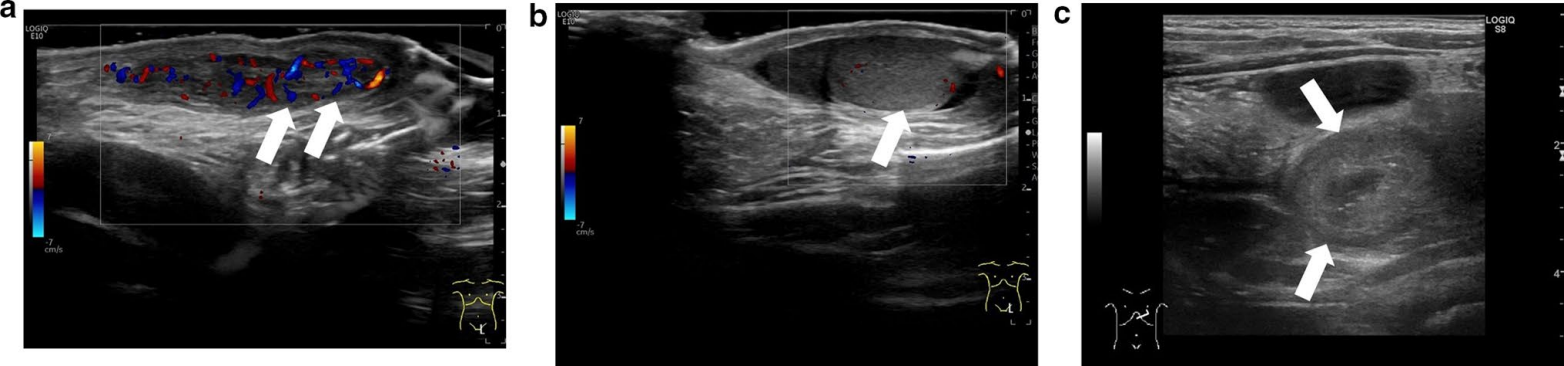
Ultrasonographic images of a 4-year-old with left acute scrotum with IgA vasculitis. **a** Swelling of the left epididymis and increased vascularity are evident. **b** Vascular flow within the left testis is preserved. **c** Wall thickening and edematous changes are evident in the dermis of the descending duodenum of the right and left scrota. Purpura was detected, leading to the diagnosis of IgA vasculitis

**Fig. 13:**
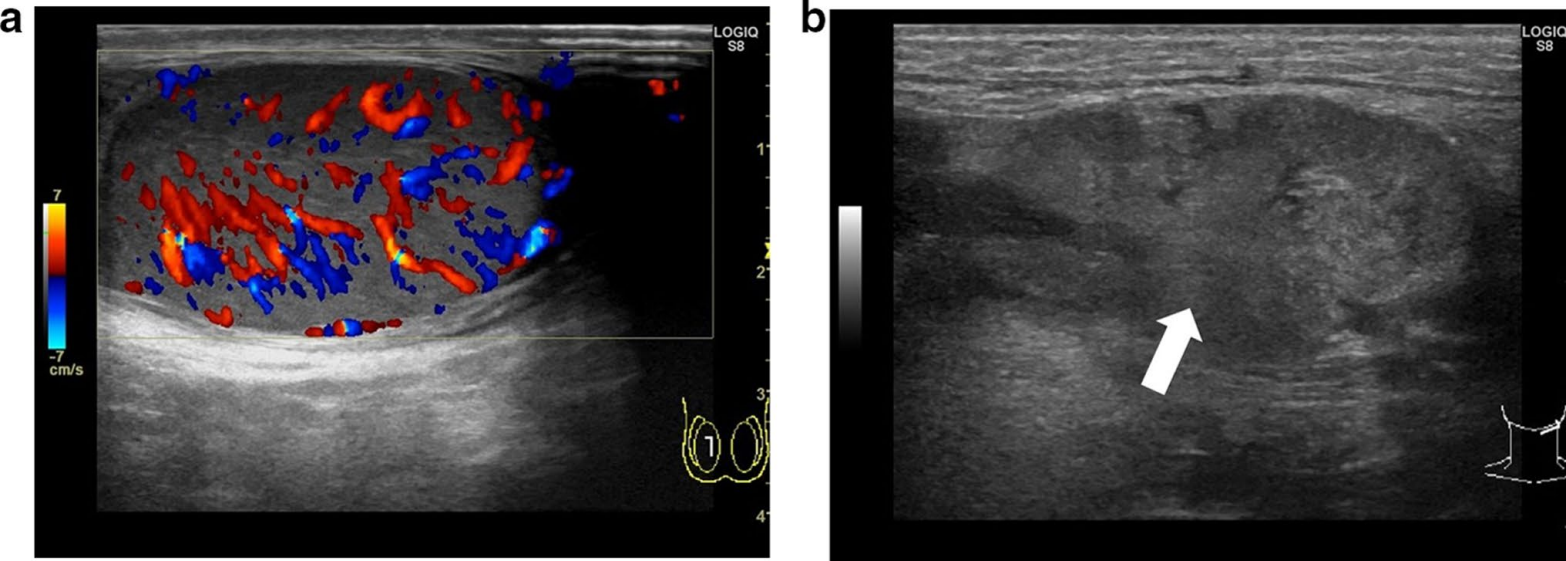
Ultrasonographic images of a 11-month-old boy with orchitis due to mumps infection. **a** The right testis is enlarged. Color Doppler sonogram shows hypervascularity within the testis. **b** The submandibular gland is swollen. Orchitis due to mumps infection was diagnosed based on these findings

Patients with acute scrotum may have various comorbidities. Idiopathic scrotal edema or epididymis may occur in patients with IgA vasculitis or Kawasaki disease [54–57]. Acute scrotum may be the first symptom of a systemic disease; therefore, gaining knowledge regarding the diseases associated with acute scrotum is necessary. Occurrence of orchitis following mumps has been reported, and it may result in the loss of fertility [58]. Figures 11, 12, and 13 present representative images depicting idiopathic scrotal edema or orchitis associated with systemic diseases.

(5) Torsion of the epididymal appendix

**Fig. 14:**
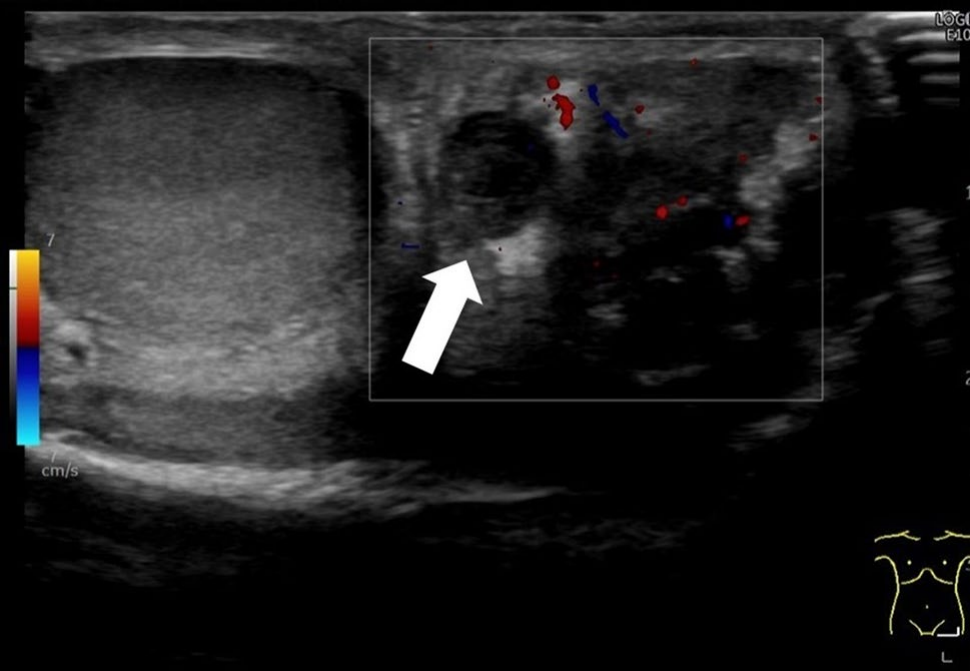
Ultrasonographic images of a 15-year-old boy with torsion of appendix epididymis. Ultrasonography shows the presence of a low-echoic mass adjacent to the testis. Color Doppler ultrasonography shows absence of blood flow within the low-echoic lesion. A diagnosis of torsion of the appendiceal testis was made

The clinical presentation of torsion of the epididymal appendix is similar to that of testicular torsion in acute scrotum [59, 60]. The appendix testis is a remnant of the Müllerian duct, whereas the epididymal appendix is a remnant of the Wolffian duct [4, 25]. The characteristic ultrasonographic findings are twisting of the appendix with an increase in size and hyperechogenicity [4, 60]. These conditions usually do not require surgical intervention and are self-limiting. Figure 14 shows representative images depicting torsion of the epididymal appendix.

(6) Testicular trauma

**Fig. 15:**
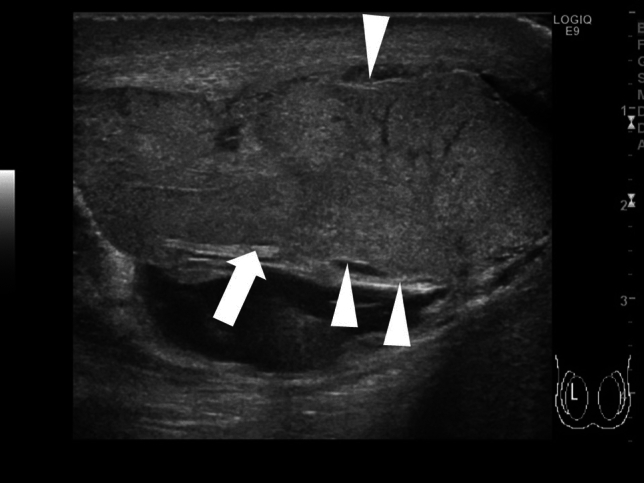
Ultrasonographic images of a 14-year-old boy with right testicular trauma and testicular rupture. The tunica albuginea is damaged in the upper pole

Testicular trauma is a common cause of acute scrotal pain [61]. Preservation of the tunica albuginea plays a crucial role in the management of testicular trauma. Testicular rupture is defined as disruption of the tunica albuginea, and surgical intervention is usually recommended in these cases [61–64]. The tunica albuginea is normally visualized as a smooth echogenic line on ultrasonography, and its disruption indicates testicular rupture [1, 59, 61, 64, 65]. Figure 15 presents representative images depicting testicular trauma.

### Useful information for manual detorsion using ultrasonography

#### Diagnosis of the directionality of testicular rotation and the degree of spermatic cord twist

The directionality of testicular rotation and degree of spermatic cord twisting can be diagnosed based on sonographic findings [17–19].

1. Direction of testicular torsion

**Fig. 16:**
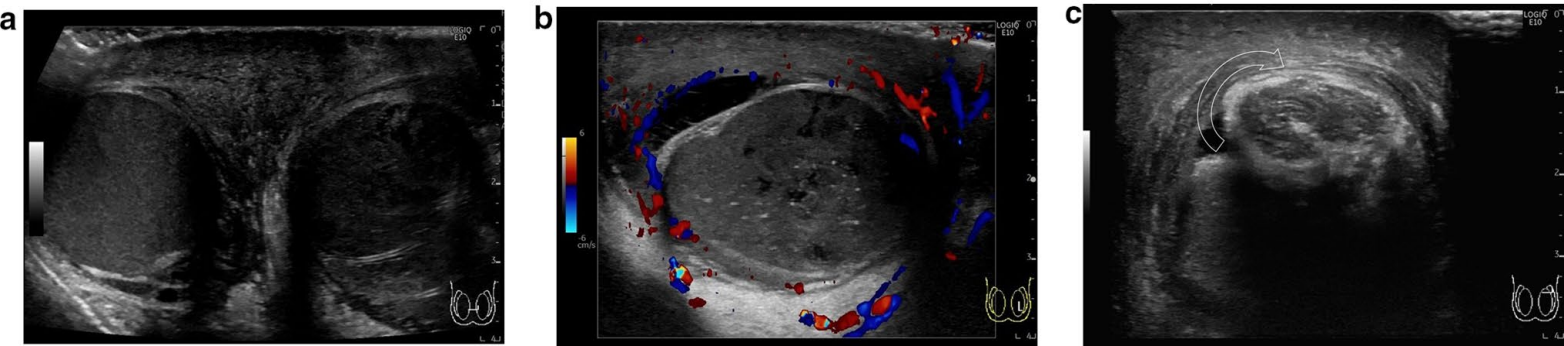
Ultrasonographic images of a 13-year-old boy with testicular torsion. **a** The left testis is enlarged and shows heterogenous echogenicity. **b **Vascular flow is absent within the affected testis. **c** The spermatic cord is twisted in the outer direction. This direction is considered atypical. Surgical exploration was performed, and the outer direction of the spermatic cord twist was confirmed

**Fig. 17:**
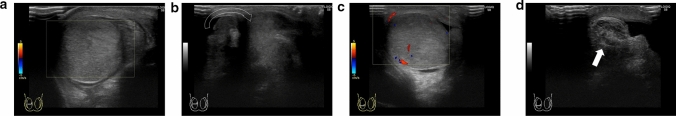
Ultrasonographic images of a 15-year-old with right testicular torsion. **a** Vascularity within the right testis is absent. **b** The whirlpool sign is evident. **c** Manual detorsion for outward twisting of the chord was performed at 360°. Hypervascularity was not evident, but the vascular flow recovered slightly. **d** The whirlpool sign is not clearly detected. Due to absence of hypervascularity, surgical exploration was performed. Residual torsion was not evident during surgical exploration. However, fasciotomy was performed due to testicular compartment syndrome

**Fig. 18:**
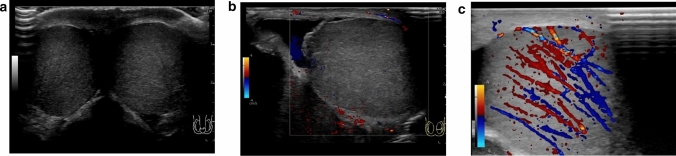
Ultrasonographic images of a 13-year-old boy with left testicular torsion. **a** The left testis has an abnormal axis. **b** Absence of blood flow within the testis is evident. **c** Outward manual detorsion by 720° is performed. Hypervascular flow within the whole affected testis is evident throughout the testis. Movie before manual detorsion; ultrasonogram shows outward twisting of the left spermatic cord (curved arrow). The spermatic cord shows 720° twisting. Manual detorsion was performed in the inward direction based on these findings

The diagnostic accuracy of ultrasonography for the direction of spermatic cord twisting has been reported to be 70% [17]. The directionality of spermatic cord twisting is first classified as counterclockwise or clockwise (viewed below) and then as inward or outward. The inward direction includes counterclockwise rotation in the left and right testes, whereas the outward direction includes clockwise rotation in the left and right testes. Figures 1, 16, 17, and 18 show representative images depicting the direction of testicular torsion.

(2) Degree of spermatic cord twisting

The degree of spermatic cord twisting is diagnosed based on the sonographic findings [17–19]. This information plays a crucial role in determining the degree of manual detorsion. The degree of manual detorsion performed varies in different cases; therefore, sonographers must carefully evaluate the degree of torsion in each examination during the procedure.

Figures 1 and 18 (movie) show representative images depicting the degree of spermatic cord twist.

#### Manual detorsion

Manual detorsion is performed by pediatric surgeons or urologists after diagnosing testicular torsion using ultrasonography. The affected testis is rotated outward (medial to lateral) or inward (lateral to medial) during the procedure based on the ultrasonographic direction of testicular rotation and the symptoms observed in each case [7, 17]. The degree of detorsion is determined based on the ultrasonographic findings and testicular pain [7, 17].

Anesthesia is not induced to prevent pain. Manual detorsion is terminated based on complete or partial pain relief. Successful manual detorsion may not result in complete pain relief on some cases; however, pain plays an important role in the assessment of the success or failure of manual detorsion [7, 10]. 

#### Ultrasonographic evaluation for determining the success of manual detorsion

Failure of manual detorsion is defined as residual spermatic cord twisting. The following ultrasonographic findings can be used to determine the success or failure of manual detorsion [13, 17, 24, 25, 27, 34, 61, 66].

1. Presence or absence of the whirlpool sign

Presence of the whirlpool sign indicates residual testicular torsion. Failure may occur due to misdiagnosis of the direction of manual detorsion, a greater degree of spermatic cord twisting, and insufficient detorsion. Therefore, the direction of the spermatic cord twisting should be carefully evaluated, and additional manual detorsion or urgent surgical exploration should be performed as necessary [7, 13]. Figures 2 and 18 (movie) show representative images.

(2) Degree and extent of perfusion within the affected testis

Only detorsion is performed during manual detorsion; orchiopexy, which prevents the recurrence of testicular torsion, and fasciotomy, which prevents testicular compartment syndrome due to reperfusion, cannot be performed during manual detorsion [16, 20, 67, 68]. Hypervascularity of the whole testis indicates successful manual detorsion. Reperfusion cannot be achieved in cases of testicular compartment syndrome, failure of manual detorsion, or testicular necrosis that has already occurred [16, 20, 67, 68]. The timing of testicular hyperperfusion after successful detorsion remains unclear; therefore, hypervascularity may be obscured even in cases with successful manual detorsion, making differentiation of focal testicular necrosis, residual testicular torsion, or testicular compartment syndrome is difficult [7, 10, 15, 16]. Urgent subsequent surgical explorations are recommended in such cases. Figures 3, 17, and 18 show representative images.

#### After manual detorsion

The success or failure of manual detorsion is determined based on the symptoms and ultrasonographic findings. Subsequent surgical exploration is usually recommended in all cases regardless of the success of manual detorsion.

## Discussion

**Fig. 19:**
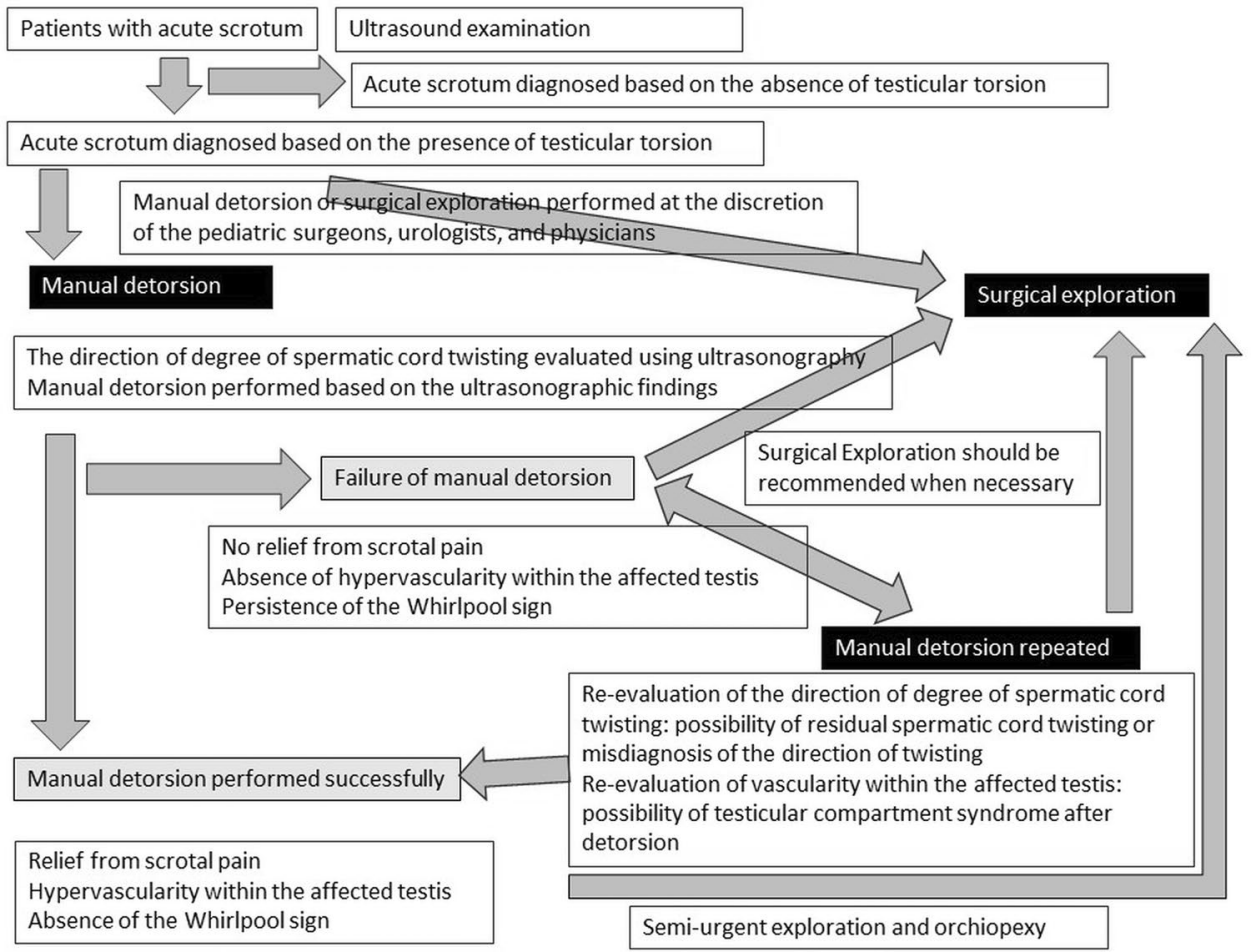
Flow chart of management of testicular torsion

This review describes the use of ultrasonography for the diagnosis and treatment of testicular torsion. The treatment strategies are summarized in Fig. 19. The diagnostic performance of ultrasonography for the detection of testicular torsion is reliable. The use of point-of-care ultrasonography for diagnosing testicular torsion by physicians in emergency departments would lead to correct diagnosis and prevention of misdiagnosis.

Identifying the direction of spermatic cord twisting is important as it determines the direction of detorsion during manual detorsion. Manual detorsion is typically performed in the outward direction first [7, 8, 10]. Physicians must perform detorsion in the outward direction if the ultrasonographic findings indicate that the direction of spermatic cord twisting is inner. However, if the ultrasonographic findings indicate that the direction of spermatic cord twisting is opposite and pain relief is not achieved via manual detorsion, physicians should consider misdiagnosis of the direction of the spermatic cord twist. Atypical direction of spermatic cord twisting, i.e., in the outward direction, reportedly occurs in 40% of cases with testicular torsion [17, 69].

Hyperperfusion, which indicates successful testicular torsion, is usually easily visualized via ultrasonography after successful manual detorsion [13, 16, 20, 21, 37]. Therefore, the most important aspect of ultrasonography during manual detorsion is determining the failure of detorsion to enable prompt transition to surgical exploration. Residual spermatic cord twisting, inadequate detorsion, presence of testicular necrosis, or testicular compartment syndrome may result in the absence of hypervascularity within the affected testis. Fasciotomy prevents testicular compartment syndrome, which may occur due to hyperperfusion of the tunica albuginea surrounding the affected testis after testicular torsion, resulting in increased venous resistance [16, 20, 67, 68]. Hyperperfusion is partially lost and not detected in the entire testis in this situation [16, 20]. Sonographers must carefully re-evaluate the direction of the spermatic cord twisting and the degree of testicular perfusion, and a suitable course of action must be determined based on these sonographic findings.

Thus, manual detorsion is a useful procedure to reduce the duration of ischemia in patients with testicular torsion. Ultrasonography can provide information regarding the direction and degree of spermatic cord twisting and aid in determining the success of manual detorsion.

### Supplementary Information

Below is the link to the electronic supplementary material.

Supplementary file1 (WMV 24197 KB)

Supplementary file2 (WMV 26478 KB)

Supplementary file3 (WMV 59877 KB)
